# Epigenetics in the Eye: An Overview of the Most Relevant Ocular Diseases

**DOI:** 10.3389/fgene.2017.00144

**Published:** 2017-10-12

**Authors:** Hanan A. Alkozi, Rafael Franco, Jesús J. Pintor

**Affiliations:** ^1^Department of Biochemistry and Molecular Biology IV, Faculty of Optics and Optometry, University Complutense of Madrid, Madrid, Spain; ^2^Department of Biochemistry and Molecular Biomedicine of the University of Barcelona, Institut de Biomedicina de la Universitat de Barcelona (IBUB), Barcelona, Spain; ^3^Cell and Molecular Neuropharmacology, Centro de Investigación Biomédica en Red Enfermedades Neurodegenerativas (CIBERNED), Instituto de Salud Carlos III, Madrid, Spain

**Keywords:** cataracts, glaucoma, dry eye, keratoconus, eye diseases

## Abstract

Sight for mammals is one of the most appreciated senses. In humans there are several factors that contribute to the increment in all kind of eye diseases. This mini-review will focus on some diseases whose prevalence is steadily increasing year after year for non-genetic reasons, namely cataracts, dry eye, and glaucoma. Aging, diet, inflammation, drugs, oxidative stress, seasonal and circadian style-of-live changes are impacting on disease prevalence by epigenetics factors, defined as stable heritable traits that are not explained by changes in DNA sequence. The mini-review will concisely show the data showing epigenetics marks in these diseases and on how knowledge on the epigenetic alterations may guide therapeutic approaches to have a healthy eye.

## Introduction

The eye in mammals is an extraordinarily specialized sensory organ. From the outward–inward direction, the eye receives light that passing through the cornea reaches the pupil; light is finally focused by the crystalline lens. When light reaches the neurosensory retina, a series of phototransduction cascades unfold to convert the photonic energy into a neural signal going from the photoreceptors to the ganglion cells, where the information travels through the optic nerve into the brain. In the central nervous system the information is processed and consciously appreciated as vision (Forrester et al., 2015).

**FIGURE 1:**
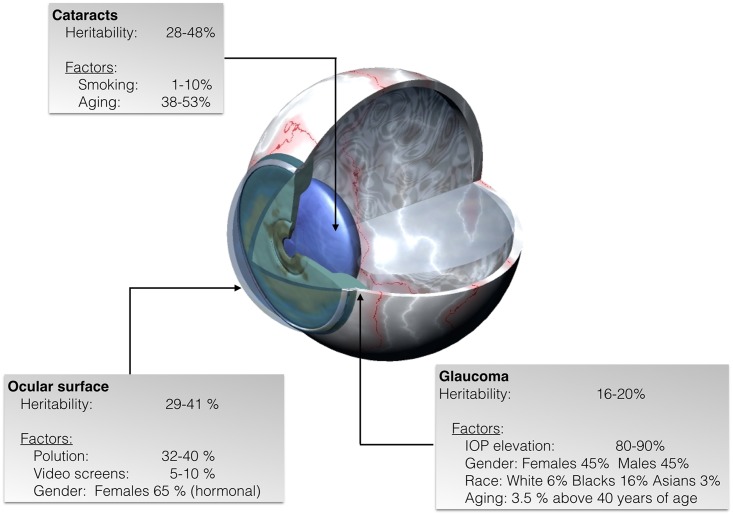
Representative scheme of eye diseases showing the estimated percentage of heritability as well as the percentage of non-inheritable factors some of which lead to specific epigenetic patterns in cataracts, glaucoma, and ocular surface diseases.

Ocular diseases and consequently, visual impairment, can occur when any cellular component of the eye becomes dysfunctional. Many ophthalmic pathologies are known to have both heritable and environmental etiopathological factors (Sanfilippo et al., 2010) (**Figure 1**). Hence, discovering the molecular mechanisms of such diseases have positive impacts in the search for therapeutic approaches (Lipinski et al., 2013; Campbell et al., 2016; Garoon and Stout, 2016). Well-powered genome-wide association and linkage studies have helped in detecting some of the causes of ocular diseases. Taking into account the human lifespan, a high percentage (circa 90%) of the genes of the human genome are expressed in eye structures (Sheffield and Stone, 2011). However, the precise reason behind developmental genetic features and regulation of gene expression by environmental factors remains poorly understood.

Gene–environment interaction dynamics are currently known to be potentially mediated by epigenetic marks established during development and/or acquired by food habits and environmental factors (Leenen et al., 2016; Hewitt et al., 2017). Limited but increasing evidence suggest an epigenetic basis in several ocular diseases (Wei et al., 2012; Busanello et al., 2017).

Epigenetics can be defined as the mitotically and mitotically heritable potential for gene expression that does not involve variation in the DNA sequence (Ushijima et al., 2003; Fedoriw et al., 2012; Waddington, 2012). Epigenetics variations have an important role in regulating key biological processes, such as cell differentiation, genomic imprinting, and X-chromosome inactivation (Fedoriw et al., 2012; Li et al., 2012; Wutz, 2013). Epigenetic traits are also directly or indirectly related to a wide range of diseases, from allergies to cancer (Rakyan et al., 2011).

The main mechanisms underlying epigenetics effects include DNA methylation, post-translational histone modification, chromatin remodeling, and RNA-associated gene regulation by non-coding RNAs (Ecker et al., 2017). Wide range of studies are focused on discovering possible epigenetic regulations in several pathologies. Although the field is in its beginnings, DNA methylation is by far the most widely studied epigenetic process, and many studies have begun to explore the link between DNA methylation patterns and a wide range of diseases (Hewitt et al., 2017).

DNA methyltransferase (DNMT) activity is increased in numerous diseases, and inhibitors of these enzymes are extensively studied as a pharmacological approach focusing on epigenetics-based anti-cancer therapy (Lyko and Brown, 2005). A proof of the potential of enzyme inhibitors involved in placing epigenetic marks in the DNA is the approval by the US Food and Drug Administration of a DNMT inhibitor, 5-azacytidine, as anti-tumor agent (see Kaminskas et al., 2005; Sampol et al., 2017, and references therein).

DNA methyltransferases are a family of enzymes that methylate DNA at the carbon-5 position of cytosine residues. Methylated DNA can then interact with methyl-binding proteins that function as adaptors between patches of methylated DNA and chromatin-modifying enzymes (e.g., histone deacetylases and histone methyltransferases). Histone-modifying enzymes then covalently modify histones to induce the formation of chromatin structures that repress gene transcription. There are two types of DNMTs inhibitors, namely, nucleoside and non-nucleoside inhibitors (Lyko and Brown, 2005). Acetylation or deacetylation of histone N-terminal ends is able to produce changes in the interaction between histones and DNA in chromatin, this chromatin remodeling being identified as a key step to regulate gene expression. Histone acetyltransferases and deacetylases are, respectively, the enzymes committed to the addition and removal of acetyl groups from lysine residues within histone N-terminal ends; they play an essential role in developmental processes, whereas they appear as dysregulated in a variety of diseases (Nusinzon and Horvath, 2005; Verdone et al., 2005). Generally, hyperacetylation is associated to transcriptional activation, while hypoacetylation is linked to silencing (Saha and Pahan, 2006).

During the last decade, technological advances applied to functional genomics have illustrated a new scenario in the field of RNA biology. To date, approximately 35% (about 57,000; GENCODE version 17) (Consortium, 2012) of the human sequences identified by the ENCODE project, include open reading frames but most of the remaining ones are known as “non-coding” RNAs. Non-coding RNAs are classified in different groups in accordance to their length, function, localization, orientation, or other criteria. Overall, their relevance is increasingly acknowledged by researchers because they may regulate gene expression thus arising as epigenetic players. Countless microRNAs have been discovered and described in the past few years (Morozova et al., 2012; Romano et al., 2017) and they are attributed far more physiological roles than expected when first discovered. There are still questions to sort out before apprehending the real relevance of non-coding RNAs. As an example some of those RNAs which are not translated into any protein may arrange into silencing (RISC) complexes that block the expression of given genes (Esquela-Kerscher and Slack, 2006). Other identified small RNA species are known as: PIWI-interacting RNAs (Li and Liu, 2011) circular RNAs (Tay et al., 2014), or telomeric RNAs (Schoeftner and Blasco, 2010). Longer RNAs include the so-called long non-coding RNAs, which are longer than 200 nucleotides and represent more than 20% of the human genome. It is yet unclear their relevance and function although evidence points to a substantial role in cell growth and apoptosis (Hung et al., 2011; Zhang and Peng, 2015), and in cell pluripotency and differentiation (Klattenhoff et al., 2013). Moreover, it is suggested that these long nucleic acids may act in cooperation with chromatin modifiers, thus becoming players in epigenetics-related regulation of gene expression (De Lucia and Dean, 2011; Kitagawa et al., 2012; Marchese and Huarte, 2014).

Several factors that result in changes at the epigenetic level also impact on the health of the eye. Such factors include aging, diet, inflammation, drugs, oxidative stress, and seasonal and diurnal changes (Handy et al., 2011; Mazzio and Soliman, 2012). In the present article we revise the epigenetic determinants of the most prevalent ocular diseases: cataracts, glaucoma, and dry eye.

## Cataracts

Cataracts are the result of opacity (clouding) in the crystalline lens, blocking the transmission of light that reaches the retina. According to the World Health Organization (WHO) it is the number one leading cause of reversible vision loss; although it is reversible by means of appropriate interventions, WHO estimates that, worldwide, cataracts are the cause of approximately 50% of the cases of blindness (Mahdi et al., 2014; Patil et al., 2014). Due to the increase of life expectancy that leads to progressive expansion of the elderly population, the National Eye Institute, estimates the number of people in the U.S. with cataract will double by the 2050, from the current 24.4 million to about 50 million cases. Cataracts may start as early as in 40 years old individuals and the risk, which increases with age, is estimated in 70% in 80-year-old caucasians and in 53 and 61% in afroamericans and hispanic americans, respectively. It is known that the eye has many protective mechanisms but they succumb upon sustained challenging by environmental stressors like electrophilic reactive species, drugs, inflammation, radiation, sunlight, and diabetes (**Figure 1**). Currently, there is not any pharmacological therapy for treatment or prevention for of cataracts; fortunately clear vision can be restored by surgical intervention and replacement of the cloudy lens by a synthetic transparent one. However, it is important to highlight complications associated to surgery and to the emotional concerns and costs derived from surgery and its potential complications.

Klotho gene family seems to be involved in the susceptibility and development of cataracts. Interestingly, differential epigenetics patterns in the DNA around these genes are found in the senile cataract (Jin et al., 2015). Klotho is a quite recently discovered gene family showing close correlation between expression and age. Klotho gene family consists of three members, α-Klotho, β-Klotho, and γ-Klotho, that encode type I transmembrane glycoproteins with extracellular β-glycosidase-like domains. Expression of these proteins appears as a factor related to the progression of age-related and chronic diseases in mammals. These proteins seemingly regulate the metabolism of several vitamins and minerals such as vitamin D and calcium and it is suggested that they have a role in immunological functions and in protecting the cardiovascular system (Arking et al., 2003; Kurosu et al., 2005; Alesutan et al., 2013) (**Figure 1**).

In studies performed on cataract models, an age-dependent increased methylation of Klotho’s gene promoters has been detected (Zhang et al., 2017). On the other hand, it is well-known that lens crystallin proteins play a crucial role in maintaining lens transparency (Andley, 2009; Christopher et al., 2014). As a major structural protein component, α-crystallin represents 35% of all crystallins in the lens (Thampi et al., 2002) and it serves as molecular chaperone to prevent aggregation of other crystallins (Horwitz, 2003). Recent studies showed a decreased level of α-crystallin in age-related nuclear cataract. The reduction of α-crystallin expression is linked with the hypermethylation of the CpG island in the CRYAA gene promoter (**Table 1**). Moreover, the treatment with DNA-methylation inhibitors results in restoring CRYAA gene expression (Zhou et al., 2012a,b). Such evidence sustains an epigenetic-based repression of CRYAA in age-related nuclear cataracts. A reasonable therapeutic approach, which is already suggested to treat cancer, would be the use of inhibitors of the methyltransferase since in this way it would be possible to treat this cause of cataracts.

**Table 1:** Ocular disorders and the corresponding altered epigenetic factors.

Disease	Epigenetic factors	Reference
Cataract	(1) Hypermethylation of Klotho’s gene promotor(2) Hypermethylation of the CpG island in CRYAA gene promotor(3) Demethylation of Keap1	Zhou et al., 2012a,b; Gao et al., 2015;Zhang et al., 2017

Glaucoma	(1) Upregulation of histone 2 and 3 deacetylase (HDAC 2 and 3)(2) Downregulation of histone H4 acetylase(3) Increase in DNA methylation	Schmitt et al., 2014; McDonnell et al., 2016

Ocular surface disorders	(1) Altered histone methylation pattern(2) Involvement of long non-coding RNAs in keratoconus	Ito, 2007; Szczesniak et al., 2017


Another epigenetic-based silencing has been reported for a nuclear factor, namely the erythroid 2-related factor 2 (Nrf2). This protein is a transcriptional activator that may protect the lens by binding to antioxidant response elements, which are *Cis*-acting enhancer sequences (*cis*-acting) in regulatory locus of genes related to detoxification. Although this suppression has been linked to aging and cataract formation, Nrf2 action negatively correlates with a protein called Keap1, whose expression increases with age. In fact, it seems that when Keap1 increases, it stimulates the proteasome-mediated degradation of Nrf2 that in turn would suppress Nrf2- dependent antioxidant protection of the lens (McMahon et al., 2003). Results of the analysis performed using age-related cataract crystalline lenses showed a significant demethylation in Keap1 and a decline in Nrf2, these results being similar to the ones found in lenses from a group of patients between 65 and 80 years of age (Gao et al., 2015).

## Glaucoma

Glaucoma refers to a wide spectrum of ocular conditions with multifactorial etiology distinguished by progressive irreversible optic neuropathy and peripheral visual field loss (Casson et al., 2012). Glaucoma is the second leading cause of vision loss, and it has been estimated that about 61 million people worldwide suffer from this disease; the number of afflicted individuals may increase to about 80 million by year 2020 (Quigley and Broman, 2006). Risk factors that contribute to glaucoma development are numerous and include increased intraocular pressure (IOP), age, and genetic mutations (Topouzis et al., 2009, 2011) (**Figure 1**). Strong evidence shows that predisposing single nucleotide polymorphisms (SNPs) and environmental effects are also key factors in the development of glaucoma (Chen et al., 2012; Guo et al., 2012).

Glaucoma courses with epigenetic alteration in several ocular structures. For instance, histone 2 and 3 deacetylase (HDAC 2 and 3) expression are significantly upregulated after acute optic nerve injury; however, histone H4 acetylase is downregulated. These data indicate that epigenetic patterns do vary upon optic nerve damage (Schmitt et al., 2014). Moreover, inhibition of retinal HDAC activity in the retina was successfully able to both, preserve the expression of a representative retinal ganglion cell-specific gene and attenuate cell loss in response to optic nerve damage (Pelzel et al., 2010). In addition, a positive effect was observed after using valporic acid (VPA) as a neuroprotective agent for injured retinae, this agent is suggested to directly inhibit HDAC activity and cause histone hyperacetylation (Phiel et al., 2001; Biermann et al., 2010). These reports revealed that abnormal histone acetylation/deacetyalation might be related to retinal ganglion cell damage in glaucoma.

A different aspect of the pathophysiology of glaucoma is the accumulation of extracellular matrix (ECM) in the trabecular meshwork, the conventional pathway of aqueous humor drainage. When the trabecular meshwork is blocked by an abnormal structure in the ECM, aqueous humor does not find a way out and it accumulates within the eye, so IOP increases. Apart from subsequent fibrosis, another relevant finding is the hypoxic environment of the trabecular meshwork of glaucomatous eyes; hypoxia leads to substantial increase in DNA methylation in locuses related to the regulation of the expression of the pro-fibrotic (TGF)β1 factor and the Ras protein activator like 1 (RASAL1) (McDonnell et al., 2016) (**Figure 1**).

Convergent evidence suggests an association between glaucoma and some genetic variants in the CDKN2B-CDKN2A gene cluster at 9p21 chromosome (Wiggs et al., 2012). CDKN2B-AS, also known as ANRIL (Antisense Non-coding RNA in the INK4 Locus), is a long non-coding RNA transcribed in the antisense direction of CDKN2B-CDKN2A (Pasquale et al., 2013). This chromosome locus is a hotspot for numerous disease related polymorphisms; in fact, ANRIL has been recently associated *inter alia* to several cancers, diabetes, and glaucoma (**Table 1**) (Congrains et al., 2013). Although the mechanism behind the association between glaucoma and ANRIL is poorly understood, it seems to be more epigenetic than genetic. Various hypothesis have been proposed to explain the link such as that occurrence of polymorphisms at this locus changes the expression of target genes responsible of cell cycle regulation, subsequently inducing retinal ganglion cell apoptosis (Burdon et al., 2011). Another study suggests that the ANRIL region is involved in regulating the vulnerability of the optic nerve subsequent to the progression of the disease (Pasquale et al., 2013).

## Ocular Surface Disorders

The surface of the eye is constantly facing external environmental stress; it contains the cornea, which is highly responsible of the refractive effect of the eye. It is highly innervated, avascular and transparent and it obtains nutrients from the aqueous humor and the tear film (Brubaker et al., 1975; Forrester et al., 2015). The ocular surface is prone to harmful events starting by tear-film disruption that may end up in a dry eye, which is a prevalent disease. Several studies have suggested the involvement of epigenetics in the pathophysiology of disorders affecting the ocular surface.

Dry eye is one of the under-diagnosed ocular surface diseases. It has become the most common ocular surface alteration worldwide and it is defined by the International Dry Eye Workshop (DEWS) as “a multifactorial disease of the tears and ocular surface that results in symptoms of discomfort, visual disturbance, and tear film instability with potential damage to the ocular surface. It is accompanied by increased osmolarity of the tear film and inflammation of the ocular surface” (International Dry Eye Workshop (DEWS) Definition and Classification, 2007).

Previous studies attributed dry eye to inflammation and the release of pro-inflammatory cytokines such as the promotion of the activity of nuclear factor kappa-light-chain-enhancer of activated B cells (NF-κB), but also dry eye may have an altered histone methylation pattern (Ito, 2007). Apart from inflammatory components, dry ocular surface is overexposed to infective agents, to environmental allergens and pollutants (**Figure 1**). Another quite common corneal disease is keratoconus, which often comes accompanied with dry eye. Keratoconus is a degenerative disorder characterized by thinning of the corneal stromal layer leading to a cornea with conical shape and consequently resulting in loss of visual capability. A very recent database has been created in order to characterize keratoconus transcriptome and to identify long non-coding RNAs which might be involved in keratoconus etiology. These results have shown that some of those non-coding RNAs could affect the expression of at least 996 genes in keratoconus patients (**Table 1**) (compared to healthy subjects). The differentially regulated genes include very relevant cellular metabolism and fate regulators such as TGF-β and SMAD9, SMAD6, TGFB3, and TGFBR1 members of Hippo/Wnt pathways (Szczesniak et al., 2017). All those have been previously associated with keeping ocular health (Morgan et al., 2013). Based on these epigenetics-based data, novel therapies are being approached and investigation on novel epigenetics mechanisms are undertaken to have a better understanding of the etiology of ocular surface disorders.

In summary, epigenetics play an important role in the physiology of numerous ocular diseases. Understanding such changes could open a novel window for therapeutical approaches in addition to current therapies. Moreover, further investigation could be helpful for early detection of pathologies with irreversible effect of vision loss such as glaucoma as well as disorders of the retina where solutions are often aiming to treat the symptoms rather than the disease itself.

## Author Contributions

HA: bibliographical search and writing; RF: organization and writing; JP: organization, writing, and figure design.

## Conflict of Interest Statement

The authors declare that the research was conducted in the absence of any commercial or financial relationships that could be construed as a potential conflict of interest.
